# Effect of intraoperative needle biopsy on the survival of nonsmall cell lung cancer patients: a propensity score matching analysis

**DOI:** 10.1007/s00595-022-02484-w

**Published:** 2022-03-03

**Authors:** Mizuki Morota, Masaaki Nagano, Naohiro Ijiri, Nobuyuki Yoshiyasu, Yoshikazu Shinohara, Yuya Nobori, Hirokazu Yamaguchi, Shun Kawashima, Masahiro Yanagiya, Chihiro Konoeda, Kentaro Kitano, Masaaki Sato, Jun Nakajima

**Affiliations:** Department of Thoracic Surgery, Graduate School of Medicine, The University of Tokyo, 7-3-1, Hongo, Bunkyo-ku, Tokyo Japan

**Keywords:** Needle biopsy, Intraoperative biopsy, Lung cancer, Lobectomy, Survival

## Abstract

**Purpose:**

It is unknown whether intraoperative needle biopsy (INB) predisposes to the postoperative recurrence of lung cancer and compromises the prognosis of these patients. We conducted this study to identify the effect of INB before lobectomy on the postoperative recurrence rate and prognosis of patients with nonsmall cell lung cancer (NSCLC).

**Methods:**

The subjects of this retrospective study were 953 patients with pathological stage I–III NSCLC who underwent lobectomy between 2001 and 2016. The patients were divided into two groups: the INB group (*n* = 94) and the non-INB group (*n* = 859). After propensity score matching (PSM), we compared the postoperative cumulative recurrence rate, recurrence-free survival (RFS), disease-specific survival (DSS), and overall survival (OS) between the two groups.

**Results:**

After PSM, 94 patient pairs were matched. The cumulative recurrence rate was significantly higher in the INB group than in the non-INB group (*P* = 0.01). The 5-year RFS rate was significantly lower in the INB group than in non-INB group (48% vs 68%), as were the 5-year DSS (76% vs 92%) and 5-year OS rates (67% vs 84%) (all *P* < 0.05).

**Conclusions:**

The findings of this analysis suggest that INB before lobectomy may increase the cumulative recurrence rate and worsen the prognosis of patients with resectable NSCLC. Thus, we believe that INB should be avoided unless a lung lesion cannot be diagnosed by another type of biopsy.

## Introduction

Intraoperative needle biopsy (INB) is a common intraoperative diagnostic tool used in lung cancer surgery. INB is performed before lung resection to diagnose lung tumors that were not or could not be diagnosed preoperatively. The advantage of INB over other biopsy methods is that palpation and visual confirmation are used to directly identify the lung tumor. Even under complete video-assisted thoracoscopic surgery (VATS), the sensitivity, specificity, and accuracy of INB are 94.3%, 87.5%, and 93.7%, respectively [1]. INB can also reduce the waiting time until operation relative to preoperative biopsies, and costs less than partial lung resection biopsy [1–3]. Some reports on needle biopsy described pleural dissemination, whereas others examined the pleural dissemination rate of INB and computed tomography (CT)-guided needle biopsy for lung cancer [3–7]. However, the effect of INB on the postoperative recurrence of and prognosis for lung cancer remains unclear. The objective of our study was to identify the effect of INB before lobectomy on the postoperative recurrence rate (including distant metastasis) and prognosis associated with non–small cell lung cancer (NSCLC) by comparing the outcomes of patients who underwent INB and those who did not.

## Materials and methods

This retrospective study was approved by the ethics committee of The University of Tokyo Hospital (Clinical Pilot Study No. 2406). We screened 1045 patients with pathological stage I–III NSCLC, who underwent lobectomy for curative resection (excluding pneumonectomy), with lymph node dissection, at the University of Tokyo Hospital between January, 2001 and December, 2016. Pathological staging of NSCLC was performed according to the seventh edition of The Union for International Cancer Control.

**Fig. 1:**
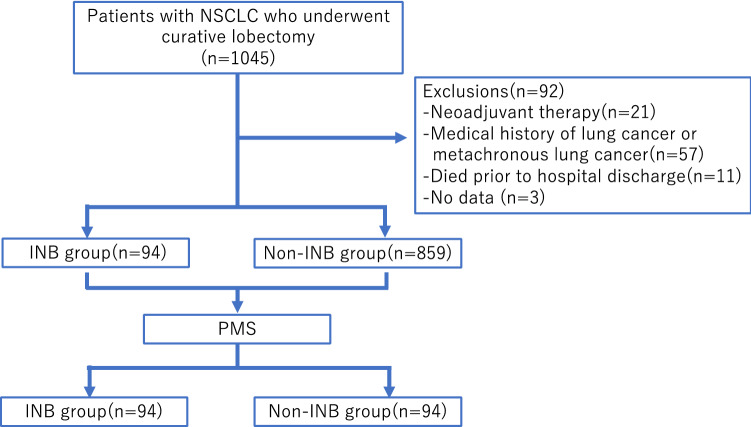
Overview of patient selection and grouping. NCSLC: non–small cell lung cancer; INB: intraoperative needle biopsy; PSM: propensity score matching

Patients who received neoadjuvant chemotherapy or radiotherapy, those with a history of lung cancer, those who had metachronous lung cancer during the observation period, those who died prior to hospital discharge, and those for whom no data were available, were excluded from the analysis (*n* = 92). Finally, 953 patients were the subjects of this retrospective study. The patients were divided into two groups: the INB group (*n* = 94) and the non-INB group (*n* = 859) (Fig. 1).

Clinical staging of NSCLC was based on the findings of CT, brain magnetic resonance imaging, and positron emission tomography. Since 2016, endobronchial ultrasound-guided transbronchial needle aspiration has been performed for suspected clinical N2 disease. A tumor was defined as central if it involved the inner one-third of the hemithorax. Lobectomy was performed by thoracotomy or VATS. ND2a-1 was performed for cN0 disease and ND2a-2 was performed for cN1 or more severe disease. During VATS lobectomy, wounds were protected by trocars or protectors, and specimens were extracted using a bag. Lung cancer was diagnosed intraoperatively in all patients in the INB group. The surgical team selected either core needle biopsy (16 G) or fine needle aspiration biopsy (18–19 G). The lung tumor was punctured several times and pressure hemostasis was applied just after the punctures. The area was washed before closing the wound. Generally, INB was performed when partial resection was made difficult by the location and size of the tumor and when the tumor was identified clearly intraoperatively and able to be punctured safely.

The non-INB group comprised patients who underwent lobectomy without biopsy, whose tumor could not be diagnosed by INB, but was diagnosed intraoperatively by partial lung resection, or preoperatively by transbronchial lung biopsy, CT-guided needle biopsy, or other methods.

The observation period was 5 years after surgery. Patients who were followed up at our hospital as outpatients underwent medical check-ups and chest X-rays or CT scans at least twice a year. The endpoints of this study were the postoperative cumulative recurrence rate, 5-year recurrence-free survival (RFS), 5-year disease-specific survival (DSS), and 5-year overall survival (OS).

### Statistical analysis

Continuous variables are presented as medians and interquartile ranges, and nominal variables are presented as numbers and percentages. All statistical analyses were performed using EZR (Saitama Medical Center, Jichi Medical University, Saitama, Japan), a graphical user interface for R (The R Foundation for Statistical Computing, Vienna, Austria, version 3.6.1) [8]. Non-normally distributed continuous variables were compared by the Mann–Whitney *U* test and categorical variables were compared by Pearson's chi-square test or Fisher’s exact test. Gray's test was used to compare cumulative recurrence rates and the Kaplan–Meier method with the log-rank test was used to compare the RFS, DSS, and OS curves between the two groups. All *p* values were two-sided, and *P* < 0.05 was considered statistically significant. Propensity scores were calculated by logistic regression using 11 baseline characteristics (gender, age, histopathology, *p*-stage, tumor size, pN, v, ly, pl, pm, adjuvant therapy). Patients were matched in a 1:1 ratio by the nearest neighbor method with the caliper value set at 0.2. Standardized mean differences (SMD) are used to show the balance of matching.

## Results

A total of 953 patients, divided into an INB group (*n* = 94) and a non-INB group (*n* = 859), were screened and analyzed retrospectively. Tables 1 and 2 summarize the patients' baseline characteristics. The median follow-up time was 60 (interquartile range, 41–60) months. Before propensity score matching (PSM), there were 42 (44.7%) patients with recurrence in the INB group vs. 157 (18.3%) in the non-INB group. The recurrence sites were the stump (4.3%), pleura (9.6%), lymph node (10.6%), lung (17%), brain (9.6%), and bone (3.2%) in the INB group and the stump (0.7%), pleura (3.1%), lymph node (5.9%), lung (5.4%), brain (2.3%), bone (2.3%), liver (1.3%), and others (0.6%) in the non-INB group, which included duplicates (Table 3). The cumulative recurrence rate was significantly higher in the INB group than in the non-INB group (*P* < 0.001). Five-year RFS was 48% in the INB group and 76% in the non-INB group, 5-year DSS was 76% in the INB group and 92% in the non-INB group, and 5-year OS was 67% in the INB group and 87% in the non-INB group. All of these differences were significant (*P* < 0.001). In the INB group, there were 29 patients who underwent core needle biopsy and 65 who underwent fine needle aspiration biopsy. The cumulative recurrence rate was not significantly different according to biopsy type (*P* = 0.85). The pleural recurrence rate was 5.3% for core needle biopsy and 4.3% for fine needle aspiration biopsy (*P* = 1.0).

**Table 1:** Baseline characteristics of the enrolled patients

	Total (*n* = 953)
*Gender, n* (%)	
Female	373 (39.1)
Male	580 (60.9)
*Age*	69.0 [62.0, 75.0]
*Hisitopathology, n* (%)	
Ad	709 (74.4)
Sq	187 (19.6)
Others	57 ( 6.0)
*p-stage, n* (%)	
IA	431 (45.2)
IB	248 (26.0)
II	154 (16.2)
III	120 (12.6)
Tumor size (mm)	25.0 [16.0, 35.0]
*pN, n* (%)	
N0	761 (79.9)
N1-3	192 (20.1)
*v, n *(%)	
Negative	567 (59.5)
Positive	386 (40.5)
*ly, n *(%)	
Negative	763 (80.1)
Positive	190 (19.9)
*pl, n* (%)	
Negative	621 (65.2)
Positive	332 (34.8)
*pm, n* (%)	
Negative	916 (96.1)
Positive	37 ( 3.9)
*Adjuvant therapy, n* (%)	
–	790 (82.9)
+	163 (17.1)
*Reccurence, n* (%)	
–	754 (79.1)
+	199 (20.9)
Follow-up time (month)	60.0 [40.9, 60.0]

Variables are presented as medians (interquartile range) unless otherwise indicated

*Ad* adenocarcinoma, *Sq* Squamous cell carcinoma, *p-stage* pathological stage, *pN* pathological N category, *v* vessel invasion, *ly* lymphatic invasion, *pl* pleural invasion, *pm* pulmonary metastasis in the primary lobe, *INB* intraoperative needle biopsy, *SMD* standardized mean differences

**Table 2:** Baseline characteristics of the cohorts before and after propensity score matching

	Before PSM				After PSM			
	Non-INB group (*n* = 859)	INB group (*n* = 94)	*P*-value	SMD	Non-INB group (*n *= 94)	INB group (*n* = 94)	*P*-value	SMD
*Gender, n* (%)								
Female	335 (39.0)	38 (40.4)	0.82	0.029	41 (43.6)	38 (40.4)	0.768	0.065
Male	524 (61.0)	56 (59.6)			53 (56.4)	56 (59.6)		
*Age*	69.0 [62.0, 75.0]	68.5 [63.0, 75.0]	0.96	0.040	70.0 [62.3, 76.0]	68.5 [63.0, 75.0]	0.651	0.002
*Hisitopathology, n* (%)								
Ad	644 (75.0)	65 (69.1)	0.38	0.136	61 (64.9)	65 (69.1)	0.769	0.103
Sq	164 (19.1)	23 (24.5)			25 (26.6)	23 (24.5)		
Others	51 ( 5.9)	6 ( 6.4)			8 ( 8.5)	6 ( 6.4)		
*p-stage, n* (%)								
IA	401 (46.7)	30 (31.9)	0.034	0.317	28 (29.8)	30 (31.9)	0.96	0.089
IB	218 (25.4)	30 (31.9)			32 (34.0)	30 (31.9)		
II	137 (15.9)	17 (18.1)			15 (16.0)	17 (18.1)		
III	103 (12.0)	17 (18.1)			19 (20.2)	17 (18.1)		
Tumor size (mm)	25.0 [15.0, 35.0]	28.0 [20.0, 37.3]	0.017	0.192	28.0 [20.0, 41.5]	28.0 [20.0, 37.3]	0.971	0.003
*pN, n* (%)								
N0	690 (80.3)	71 (75.5)	0.28	0.116	68 (72.3)	71 (75.5)	0.74	0.073
N1-3	169 (19.7)	23 (24.5)			26 (27.7)	23 (24.5)		
*v, n* (%)								
Negative	518 (60.3)	49 (52.1)	0.15	0.165	48 (51.1)	49 (52.1)	1	0.021
Positive	341 (39.7)	45 (47.9)			46 (48.9)	45 (47.9)		
*ly, n* (%)								
Negative	695 (80.9)	68 (72.3)	0.057	0.203	66 (70.2)	68 (72.3)	0.872	0.047
Positive	164 (19.1)	26 (27.7)			28 (29.8)	26 (27.7)		
*pl, n* (%)								
Negative	572 (66.6)	49 (52.1)	0.006	0.298	55 (58.5)	49 (52.1)	0.463	0.129
Positive	287 (33.4)	45 (47.9)			39 (41.5)	45 (47.9)		
*pm, n* (%)								
Negative	827 (96.3)	89 (94.7)	0.4	0.077	87 (92.6)	89 (94.7)	0.767	0.087
Positive	32 ( 3.7)	5 ( 5.3)			7 ( 7.4)	5 ( 5.3)		
*Adjuvant therapy, n* (%)								
–	717 (83.5)	73 (77.7)	0.15	0.147	72 (76.6)	73 (77.7)	1	0.025
+	142 (16.5)	21 (22.3)			22 (23.4)	21 (22.3)		
*Reccurence, n* (%)								
–	702 (81.7)	52 (55.3)			71 (75.5)	52 (55.3)		
+	157 (18.3)	42 (44.7)			23 (24.5)	42 (44.7)		
Follow-up time (month)	60.0 [42.1, 60.0]	55.8 [32.2, 60.0]			60.0 [35.9, 60.0]	55.8 [32.2, 60.0]		

Variables are presented as medians (interquartile range) unless otherwise indicated

*Ad* adenocarcinoma, *Sq* Squamous cell carcinoma, *p-stage* pathological stage, *pN* pathological N category, *v* vessel invasion, *ly* lymphatic invasion, *pl* pleural invasion, *pm* pulmonary metastasis in the primary lobe, *INB* intraoperative needle biopsy, *PSM* propensity score matching, *SMD* standardized mean differences

**Table 3:** Sites of recurrence in patients before and after propensity score matching

	Before PSM			After PSM		
	Non-INB group (*n* = 859)	INB group (*n* = 94)	*P*-value	Non-INB group (*n* = 94)	INB group (*n* = 94)	*P*-value
*Recurrence site* (%)						
Stump	0.7	4.3	0.01	1.1	4.3	0.37
Pleura	3.1	9.6	< 0.01	2.1	9.6	0.06
Lymph node	5.9	10.6	0.12	7.4	10.6	0.61
Lung	5.4	17.0	< 0.01	6.4	17.0	0.04
Brain	2.3	9.6	< 0.01	5.3	9.6	0.41
Bone	2.3	3.2	0.49	3.2	3.2	1.0
Liver	1.3	0.0	0.61	1.1	0.0	1.0
Others	0.6	0.0	1.0	1.1	0.0	1.0

*INB* intraoperative needle biopsy, *PSM* propensity score matching, *SMD* standardized mean differences

**Fig. 2:**
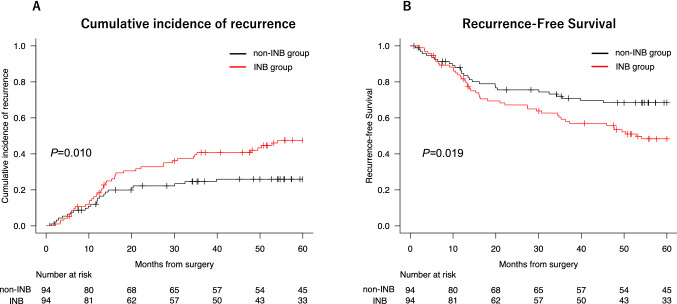
**A** Gray's test for the cumulative recurrence rate in the intraoperative needle biopsy (INB) group vs. the non-INB group (*P* = 0.010). **B** Log-rank test for 5-year recurrence-free survival (RFS) after propensity score matching. The 5-year RFS was 48% in the INB group and 68% in the non-INB group (*P* = 0.019)

**Fig. 3:**
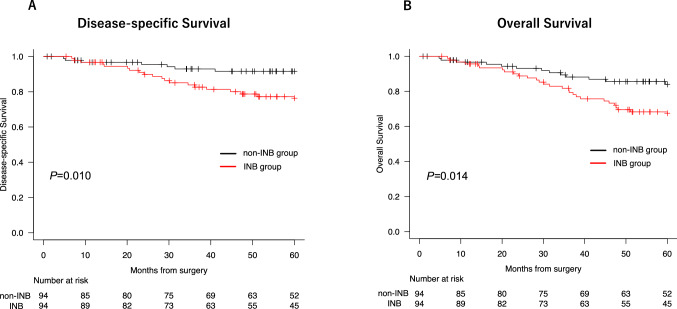
**A** Log-rank test for 5-year disease-specific survival (DSS) in the INB group vs. the non-INB group. **B** The 5-year overall survival (OS) after propensity score matching. The 5-year DSS was 76% in the INB group and 92% in the non-INB group (*P* = 0.010), and the 5-year OS was 67% in the INB group and 84% in the non-INB group (*P* = 0.014)

After PSM, 94 pairs were matched between the INB group and the non-INB group. The two groups were well matched for 11 baseline characteristics. The cumulative recurrence rate was significantly higher in the INB group than in the non-INB group (*P* = 0.01). The 5-year RFS was 48% in the INB group and 68% in the non-INB group, the 5-year DSS was 76% in the INB group and 92% in the non-INB group, and the 5-year OS was 67% in the INB group and 84% in the non-INB group. All these differences were significant (*P* < 0.05) (Figs. 2 and 3). There were no significant differences in tumor location between the groups (*P* = 0.57): 16% of tumors were central in the INB group and 20% of tumors were central in the non-INB group.

Among patients with p-stage I disease, the cumulative recurrence rate was higher in the INB group than in the non-INB group before and after PSM (*P* < 0.01). The rate of pleural recurrence was higher in the INB group than in the non-INB group before and after PSM (4.3% vs. 1.6% and 4.3% vs. 0%, respectively).

## Discussion

Based on the findings of this study, INB increased the recurrence rate and decreased 5-year survival. Sawabata et al. reported that fine-needle aspiration biopsy causes mechanical exfoliation of tumor cells into the pleural cavity [6]. During INB, blood spreads into the thoracic cavity from the puncture, forming a hematoma around the punctured spot, suggesting that the puncture causes tumor cells to become liberated, spread out, and circulate. Accordingly, in the present study, the pleural recurrence rate was higher in the INB group than in the non-INB group.

A recent study found that surgical procedures and manipulation affect the level of circulating tumor cells (CTCs) and ultimately, the prognosis. CTCs become more abundant after surgical manipulation, and patients with cluster CTCs had significantly lower RFS and OS [9–14]. Wei et al. showed that the vein-first ligating technique was better than the artery-first technique for lobectomy, in terms of prognosis and reduction of CTCs, for patients with NSCLC [15]. Yasukawa et al. found that partial resection before lobectomy decreases the recurrence rate and improves RFS of patients with early-stage lung adenocarcinoma [16]. Partial resection was used to create a tumor-free environment before lobectomy. When performing INB, the tumor remains until lobectomy is initiated. Therefore, unless the drainage pulmonary vein is ligated, CTCs will remain high. In our study, the rates of recurrence in the lung and brain were significantly higher in the INB group than in the non-INB group, supporting this theory of previous studies. Based on the nature of the INB procedure, it is easy to interpret the findings of this study.

In comparing the preoperative and intraoperative biopsies, Taniguchi et al. reported that a preoperative biopsy did not worsen prognosis relative to a lung resection biopsy in patients with cTanyN0M0 NSCLC, as revealed by multivariate analysis [17]. Conversely, Nakajima et al. reported that the RFS of patients who underwent resection of early-stage NCSLC was better in their intraoperative biopsy group than in their transbronchial biopsy group [18]. Huang et al. reported that preoperative biopsy was an independent factor influencing overall recurrence and that it worsened the disease-free survival of patients with stage I adenocarcinoma, as determined by multivariate analysis adjusted by PSM [19]. As mentioned, Yasukawa et al. reported that intraoperative partial pulmonary resection before lobectomy decreased the recurrence rate and improved RFS of early-stage lung adenocarcinoma patients [16].

Based on these studies and our findings, intraoperative partial resection biopsy seems to be a better choice for diagnosing lung tumors. However, all these studies were retrospective and the relationship between biopsy and prognosis has not been investigated fully, so it is difficult to establish which biopsy procedure is most appropriate.

When performing biopsies other than INB has been difficult and malignancy is strongly suggested by preoperative examination, complete resection without biopsy remains an option. In 2018, the 30-day mortality rate of patients who underwent lobectomy for primary lung cancer in Japan was only 0.2% [20]. Furthermore, image diagnosis supported by artificial intelligence and deep learning is being studied to improve the accuracy of preoperative noninvasive diagnosis [21–23]. If the accuracy of preoperative, noninvasive diagnostic tools is upgraded and supported by sufficient evidence, curative resection without biopsy may become a standard procedure for resectable NSCLC. If this evolves, fewer patients will require INB, potentially changing the prognosis of resectable NSCLC.

## Limitations

Our study has several limitations. First, it was a retrospective study in a single-institution setting. INB is commonly selected to diagnose peripheral solid tumors, so pl, ly, v, and other data were included as PSM characteristics to minimize selection bias, but this approximation is limited. Second, the sample size was small, with only 94 patient pairs included in the analysis. Third, we did not examine the subtype of adenocarcinoma, or the relationship between the INB procedure and CTCs. Prospective studies that examine the effect of each type of biopsy on patients with resectable NSCLC, or even retrospective studies with larger sample sizes or multicenter studies, are needed.

## Conclusions

Performing INB before lobectomy has the potential to increase the cumulative recurrence rate and worsen the prognosis of patients with resectable NSCLC. Based on our findings, INB should be limited to when the diagnosis of NSCLC cannot be confirmed using any other kind of biopsy.

## Abbreviations

INB: Intraoperative needle biopsy
VATS: Video-assisted thoracoscopic surgery
CT: Computed TOMOGRAPHY
NSCLC: Non-small cell lung cancer
RFS: Recurrence-free survival
DSS: Disease-specific survival
OS: Overall survival
SMD: Standardized mean differences
PSM: Propensity score matching
CTCs: Circulating tumor cells
Meeting presentation: Presented at the 38th Annual Meeting of the Japanese Association for Chest Surgery, Nagasaki, Japan, 20–21 May 2021.
